# The power of belief? Evidence of reduced fear extinction learning in Catholic God believers

**DOI:** 10.3389/fpubh.2024.1509388

**Published:** 2025-01-07

**Authors:** Carmelo Mario Vicario, Laura Culicetto, Chiara Lucifora, Francesca Ferraioli, Simona Massimino, Gabriella Martino, Francesco Tomaiuolo, Alessandra Maria Falzone

**Affiliations:** ^1^Dipartimento di Scienze Cognitive, Psicologiche, Pedagogiche e Degli Studi Culturali, Università di Messina, Messina, Italy; ^2^Dipartimento di Filosofia e Comunicazione, Università di Bologna, Bologna, Italy; ^3^Dipartimento di Medicina e Clinica Sperimentale, Università degli Studi di Messina, A.O.U. “G. Martino”, Via Consolare Valeria, Messina, Italy

**Keywords:** God belief, religious ideology, Pavlovian fear conditioning, reduced fear extinction learning, weaker inhibitory learning process

## Abstract

Religious beliefs can shape how people process fear. Yet the psychophysiological mechanisms underlying this phenomenon remain poorly understood. We investigated fear learning and extinction processes in a group of individuals who professed a belief in God, compared to non-believers. Using a virtual reality Pavlovian fear conditioning/extinction task, we measured neurovegetative activity associated with these forms of associative learning. Our finding shows reduced fear extinction among God believers, compared to non-believers. This suggests that the general mechanism of fear extinction learning is suppressed in these individuals. Importantly, this effect was not explained by state or trait anxiety scores. These findings align with previous evidence linking religiosity and spirituality with the neural circuit of fear and suggest that religiosity may be associated with weaker inhibitory learning processes related to fear.

## Introduction

Beliefs are mental representations that describe causal relationships between events or stimuli. One of the most influential and widely held beliefs worldwide is the belief in the existence of God. According to the buffering theory, belief in God and, therefore, with possibility of an afterlife helps alleviate the fear of death, by offering reassurance that death does not mark the end of conscious experiences (1, 2). This is particularly evident in Christian theology, where death takes on a series of positive connotations, such as a transition to eternal life and resurrection. Such beliefs provide comfort and assurance, helping individuals cope with uncertainties of life and the mysteries surrounding death. Moreover, belief in God can provide a sense of security and comprehension when confronting fears rooted in the uncertainties of existence.

Religiosity often serves as a source of comfort and a means of coping with fear and anxiety (3), suggesting that belief in God may act as a mechanism to mitigate fear. However, this belief can also function as a catalyst for fear. In many religious traditions, the concept of “fear of God” is frequently emphasized. For some, religious motivation stems from the fear of “divine punishment” or the consequences of failing to adhere to religious laws. Fear of hell, damnation, or negative judgment in the afterlife can be a powerful motivator for compliance with religious teachings. Additionally, religiosity may be linked to a fear of sin, which in turn can contribute to heightened stress. This connection is supported by Winterowd et al. (4) who found a positive relationship between perceived stress and spirituality, as measured with a standard scale.

In summary, the relationship between belief in God and fear is complex and multifaceted, functioning both as a source of comfort in fearful situations and as a potential amplifier of fear. Considering the significant role of fear in various psychopathological conditions such as, phobias, obsessive compulsive disorders, eating disorders and posttraumatic stress disorder [for a review see (5–7)], further research is crucial. Investigating this topic in greater depth will provide valuable insights into the psychophysiological mechanisms underlying this relationship, contributing to a more comprehensive understanding of its implications for mental health.

We contribute to this discussion by providing new insights using the Pavlovian fear conditioning/extinction task (8), a widely recognized and well-established protocol for experimentally investigating fear processing (9, 54). This paradigm is relevant for studying the link between religiosity and fear processing, as it allows for the measurement of autonomic response system (neurovegetative activity), which reflect the physiological response to stress, during two distinct types of associative—fear related—learning. The conditioning phase involves associating a neutral (e.g., visual) stimulus (CS+) with an aversive event (unconditioned stimulus—US), thereby is conditioning the stimulus to be perceived as fearful. The extinction phase, refers to a process through which the conditioned fear response diminishes or disappears over time when the feared stimulus is repeatedly presented without the aversive outcome that originally triggered the fear response [see also (10)].

If successful, individuals no longer exhibit the previously learned conditioned response following the extinction session. Interestingly, the relevance of the Pavlovian fear conditioning/extinction protocol in the study of the link fear/religiosity is further highlighted by a recent study by Ferguson et al. (11). This research documented a specific relation between religiosity/spirituality and a specific brain map focused on the Periaqueductal Gray (PAG), a brainstem region known for its role in fear conditioning (12).

The relevance of the PAG in fear processing has also been documented by Watson et al. (13), who identified extinction-susceptible and extinction-resistant cells within this area, believed to play a role to the persistence of fear memories following extinction. Prospectively, two scenarios are equally possible. On one hand, evidence suggesting that religiosity enhances coping strategies (3), a critical component of exposure therapy (3), and a factor known to promote fear extinction [e.g., (14)], leads us to hypothesize reduced fear conditioning (or improved fear extinction learning) in individuals who believe in God compared to those who do not (controls). On the other hand, consistent with in line with neural evidence linking religiosity with the fear conditioning/extinction brain network (11), we predict an amplified fear conditioning response (and/or reduced fear extinction learning) in individuals who believe in God compared to controls.

This latter scenario also aligns with evidence showing a link between religiosity and perceived threat (50), and results by Bullock et al. (15) indicating that religiosity may amplify perceived threat under certain circumstances. Given the equal plausibility of these scenarios, this study provides an exploratory investigation into this field. Additionally, considering the importance of anxiety in the Conditioning, generalization, and persistence of conditioned fear (9, 59), we also explored the role of anxiety in the fear conditioning of God believers. Religious belief has shown complex associations with anxiety [for a review, see (16)], which is itself a critical factor in fear conditioning (9).

## Methods

Fifty-three participants (26 Catholic God believers, 12 males, and 27 non-God believers, 12 males) were examined with a Pavlovian fear conditioning protocols used in previous investigations (17, 56). The sample size was established through a priori power analysis using G^*^Power. Assuming a conservative scenario, the analysis indicated a required total sample size of ~54 participants (27 per group) considering the study design including two groups (God believers, no believers), 2 stimuli (CS+, CS-) and 3 conditions (habituation, Conditioning, extinction), with measurements taken within subjects, to detect a medium effect size (f = 0.25) with an alpha level of 0.05 and a desired power level of 0.80. All participants were students recruited from the University of Messina. All individuals were white Caucasian, and without a history of mental disease, according to their self-assessment. Like previous investigations in the field (18) participants provided information about their belief in God by simply responding “yes” or “no” to the question: “*Do you believe in the existence of God?*” They also clarified to be Catholics.

Written informed consent was obtained from all participants before inclusion, and the protocol was approved by the local ethics committee of the Department of Cognitive, Psychological, Educational, and Cultural Studies (Approval n. COSPECS_4_2021; COSPECS_07_2022), University of Messina, Italy. The experimental procedures were conducted according to the Declaration of Helsinki principles and subsequent updated versions (19). These data are secondary to a main project exploring the link between ideological thinking and fear processing. Descriptive details are provided in the Table 1.

**Table 1:** Descriptive statistics of the variables of interest for individuals who believe and do not believe in God.

	**Age**	**Gender**	**STAI-Y1**	**STAI-Y2**
Believers	M = 23.4	M = 12	M = 40.48	M = 46.96
	SD = 3.88	F = 14	SD = 12.98	SD = 9.44
Non-believers	M = 22.29	M = 12	M = 38.11	M = 46.44
	SD = 2.87	F = 15	SD = 11.08	SD = 9.66

STAI-Y1, State Trait Anxiety Inventory, Form Y, Subscale 1; STAI-Y1, State Trait Anxiety Inventory, Form Y2, Subscale 2.

## Instruments

### State-trait anxiety inventory

To examine the relationships between anxiety and fear conditioning and extinction, we employed both the state (STAI-Y1) and trait (STAI-Y2) anxiety subscales of the State-Trait Anxiety Inventory [STAI; (20)]. We used the Italian version of the STAI, which was created by Pedrabissi and Santinello (21). This inventory comprises 20 items for assessing state anxiety and 20 items for assessing trait anxiety. Participants responded to each item on a 4-point scale ranging from 1 (“Almost Never”) to 4 (“Almost Always”). The total score for each subscale ranges from 20 to 80 points. An illustrative item from the Italian version is: “mi sento bene” (I feel well). The internal consistency for the state and trait anxiety subscales is robust, with Cronbach's alpha coefficients reported between 0.91–0.95 and 0.85–0.90, respectively.

### Virtual reality Pavlovian fear conditioning/extinction task

To study fear conditioning and extinction, we used a Pavlovian fear conditioning protocol previously created by our group (22). This task improves upon traditional non-VR-based paradigms by addressing key limitations, such as inconsistencies in eliciting universal fear responses and challenges with stimulus calibration. By utilizing a simple, controlled environment, the protocol minimizes external influences and ensures precise trial replication, effectively addressing concerns tied to the “replication crisis” (23). Furthermore, unlike classical paradigms that rely on electrical shocks, the VR-based approach eliminates discomfort and enhances ecological validity by examining fear responses in realistic scenarios. These advancements collectively enhance the accuracy, applicability, and interpretability of fear conditioning studies [for further insights on this topic refer to Lucifora et al. and Nucera (24, 25)]. Our experimental setup used the Unity engine 3D and the Oculus Rift, a VR headset equipped with two Pentile OLED displays, each with a resolution of 1,080 × 1,200 pixels, a 90 Hz refresh rate, and a 110° field of view. The device also includes position tracking, rotation capabilities, and integrated headphones that provide a 3D sound effect (26, 27). We used a graphics workstation with an NVIDIA Titan X graphics card to run simulation, ensuring consistent high-resolution rendering of the virtual environment projected to the VR headset.

Our VR paradigm, previously utilized successfully in our group's research [e.g., (17, 57)] comprised three sessions: *habituation, Conditioning*, and *extinction*. The protocol involved two differently colored doors as stimuli. During the *habituation* session (duration about 4 min), the participants viewed the two different doors (blue and red) in a total of eight trials (i.e., four trials for each door) presented in a randomized order. Each door was individually displayed for 12 s in a random order; the door remained closed for the first 9 s after it appeared and then opened for the following 3 s. No stimuli were presented while the door was open in this phase. The inter-trial interval varied between 6 and 20 s.

**Figure 1:**
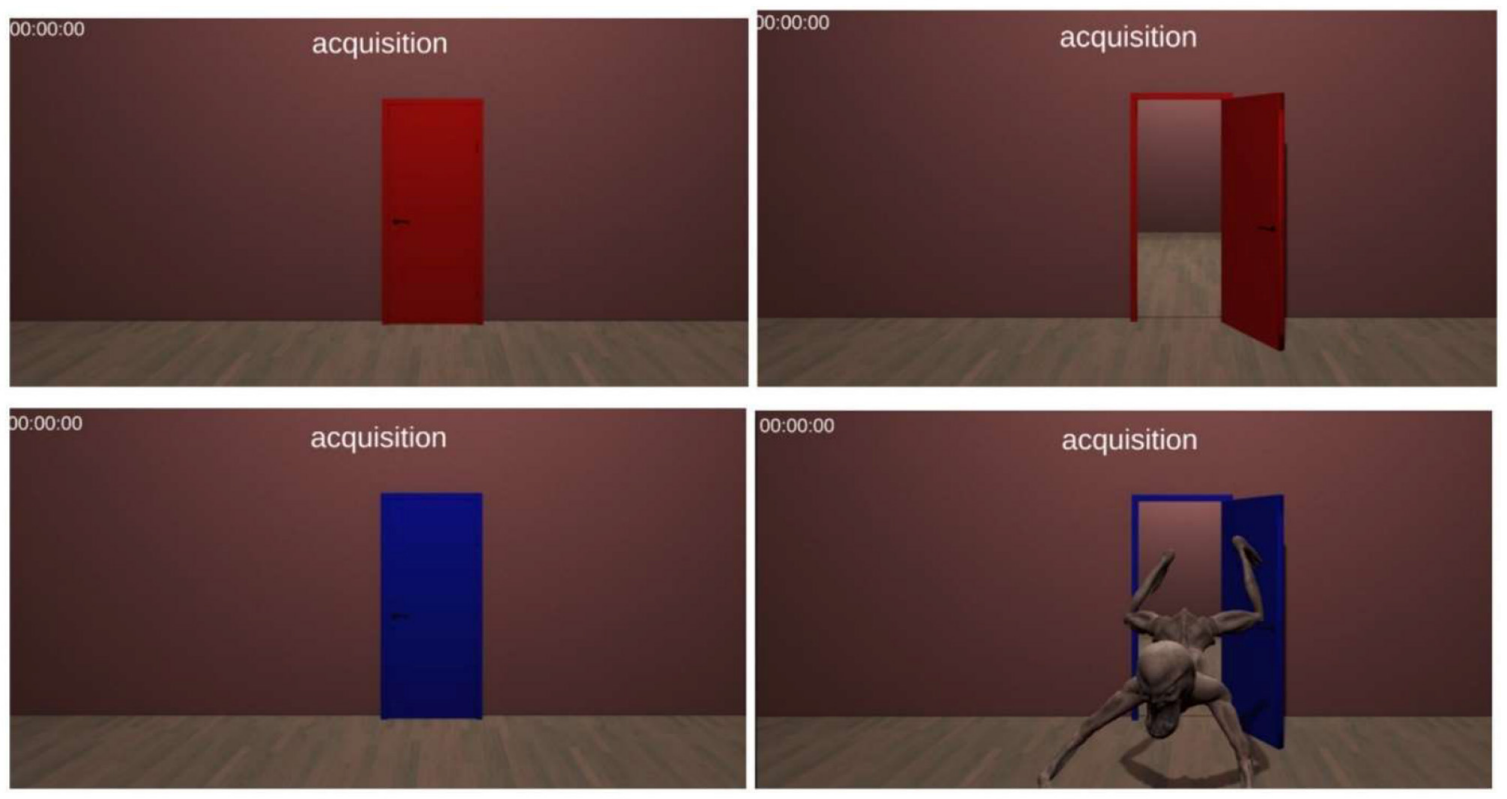
A screenshot from the conditioning phase. The upper panels depict the CS– (red door), while the lower panels show the CS+ paired with the US (blue door with the fearful stimulus). Each door remains closed for 9 s and opens for 3 s during the task.

After a 60-s break, participants proceeded to fear conditioning stage (~10 min) when they were ready. Each door was presented for a total of 12 s per trial. During the first 9 s, the door remained closed. In this session, the blue color served as the conditioning stimulus (CS+). It was paired with a threatening stimulus (a monster) in 80% of trials (eight out of 10), acting as the unconditioned stimulus (US). The US appeared during the final 3 s of the trial, immediately after the door opened. The monster then jumped toward the participant and screamed (80 dB) to elicit a fear response. The red door, in contrast, was not paired with the US after the door opened and served as the safety signal (CS-; Figure 1). The conditioning session comprised two blocks: early and late Conditioning lasting ~10 min in total.

The extinction phase, aimed at extinguishing the conditioned fear response. In this phase, the doors were again presented for a total of 12 s per trial, following the same structure: 9 s with the door closed and 3 s with the door open. The blue door (CS+), when open, was no longer paired with the US (i.e., the jumping and screaming monster), ensuring the CS+ (closed blue door) was dissociated from it over repeated trials. The extinction session comprised two blocks: early and late extinction lasting ~10 min in total. This session included 20 trials with the blue door and the red door each, presented in randomized order, similar to the conditioning phase. For conditioning and extinction phases, the closed-door represents the CS, while the open-door serves as the outcome. Both phases together create the temporal structure necessary for effective Pavlovian conditioning.

### Skin conductance response

During the task, skin conductance response (SCR) was measured using the eSense device (Mindfield Biosystems, Inc. Berlin. Germany) connected to a MEIZU M5C M710H. Electrodes were attached to the middle and index fingers with Velcro straps and connected to the device via the audio connection input. The eSense recorded data at a sampling rate of 5 Hz, which were then exported as csv files via email. SCR for the CS+ and CS– was calculated in microSiemens (μS) by subtracting the mean SCL during the 2 s prior to stimulus onset from the maximum SCL during the 12 s of stimulus presentation (3 s with the door closed and 9 s with the door open). Data extraction was performed using a MATLAB script developed in our lab specifically for this purpose.

### Fear stimulus rating

At the end of each session, participants were asked to rate the perceived level of fear associated with the presented stimuli (i.e., the red and the blue doors). This fear stimulus rating (FSR) was assessed using a 10-point Likert scale, with 1 represented “not scary at all” and 10 indicated “extremely scary.”

## Procedure

### Timeline of the experimental procedure

After participants provided informed consent, they completed the STAI-Y1, the STAI-Y2 questionnaires. They were then connected to the GSR Amp (eSense), with two ring-shaped skin conductance electrodes placed over the middle and index fingers of the right hand to measure SCR. Next, the VR helmet (Oculus Rift) was placed on the head, and the fear conditioning/extinction task was carried out. During the experimental sessions, we also collected fear stimulus ratings (FSR) to assess/monitor contingency awareness. At the conclusion of the experiment participants were debriefed and asked to indicate their belief in the existence of God by responding with either “yes, I believe in the existence of God” or “no, I do not believe in the existence of God.”

## Data analysis

Statistical analyses were conducted to test the hypothesis of a difference in fear conditioning and extinction learning between individuals stating to believe and individuals not believing in God. The SCR amplitude was determined off-line by subtracting baseline SCR (measured 2 s prior to CS presentation) from the peak skin conductance level during each CS presentation. This calculation was performed individually for each participant. To analyze the SCR data a square root transformation was applied to reduce variability, following the methodology used in previous studies of our group [e.g., (17, 28, 56)].

Separate ANOVAs involving SCR as dependent variable were conducted for each session (habituation, Conditioning, extinction) in line with previous investigations in the field [e.g., (56)]. Data were analyzed using separate mixed-model ANOVAs with a 2 (Groups: God believers, not God believers) × 2 (Stimuli: CS+, CS-) × 2 (Block: early, late) x Trials (the number of trials varied between stages) design. FSR were analyzed using a mixed model ANOVA with a 2 (Groups: God believers, not God believers) × 3 (Sessions: habituation, Conditioning, extinction) x 2 (Stimuli: CS+, CS-) factorial design. In cases of significant ANOVA results, *post-hoc* comparisons were conducted using Bonferroni-corrected *t*-tests to control for Type I errors (false positives). Effect sizes for the mixed-model ANOVAs are reported as partial-eta squared ($\eta_{p}^{2}$). *Correlation analyses*, either parametric or non-parametric, depending on the data distribution, were performed to investigate associations between the variables of interest (STAI, SCR, FSR) for each session separately. For the correlation analyses involving SCR and FSR, we calculated the differential mean (CS+ minus CS-) for each condition and session. A significant threshold of α= 0.05 was applied. Statistical analyses were performed using STATISTICA (StatSoft. Inc., Tulsa, OK, USA) version 7.0.

## Results

### Demographics

No significant difference was found for gender (*X*^2^ = 0.074, *p* = 0.784), age [*t*_(52)_ = 1.322, *p* = 0.192], STAI-Y1 [*t*_(52)_ = 0.721, *p* = 0.473] and STAI-Y2 [*t*_(52)_ = 0.199, *p* = 0.842] when comparing individuals who believe and non-believe in God.

### Skin conductance response

#### Habituation

A significant main effect of the factor trials was found [*F*_(3, 156)_ = 17.51, *p* < 0.001 η*p*^2^ = 0.252]. No further significant results were identified for habituation (refer to Supplementary Table 1 for details).

#### Conditioning

**Figure 2:**
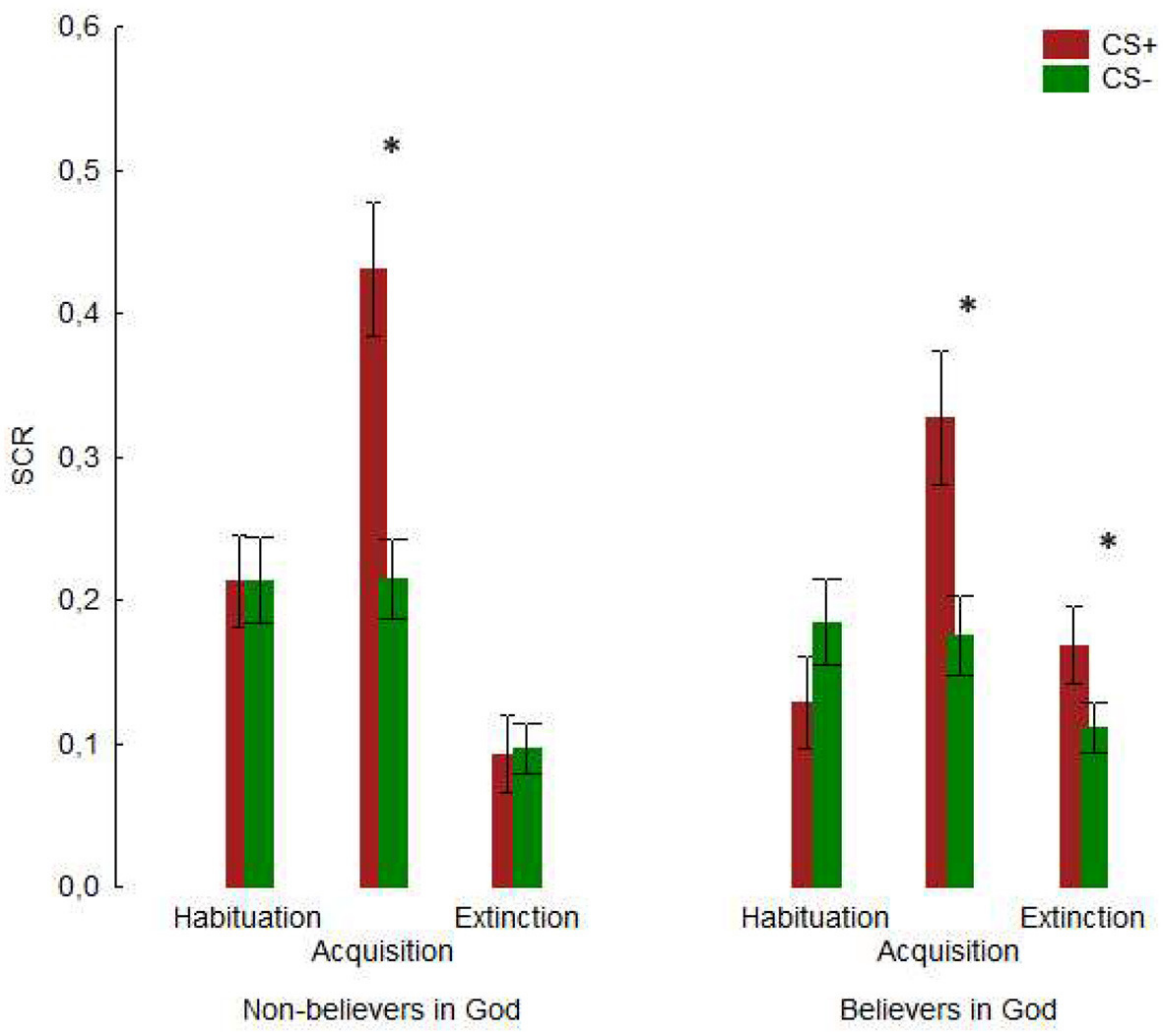
The mean SCR for CS+ and CS- during habituation, conditioning, and extinction sessions is shown for participants who state they believe in God and those who do not. An asterisk (*) indicates significant results. Vertical bars denote the standard error of the means.

We identified a significant main effect of the factor CS [*F*_(1, 52)_ =52.35, *p* < 0.001 η*p*^2^ = 0.252], with higher score for the CS+ condition (M = 0.387, SE = 0.032) compared to CS- (M = 0.210, SE = 0.018. More details on this pattern of results are provided in Figure 2). Furthermore, the main effect of the factor Block was significant [*F*_(1, 52)_ = 13.91, *p* < 0.001 η*p*^2^ = 0.211], with higher scores in the early (*M* = 0.325, *SE* = 0.024) compared to the late block (*M* = 0.272, *SE* = 0.023). No further significant results were identified for Conditioning (refer to Supplementary Table 2 for details).

#### Extinction

The factor Group x Stimulus was significant [*F*_(1, 52)_ = 4.58, *p* = 0.036, η*p*^2^ = 0.081]. *Post-hoc* comparison shows a significant difference between CS+ (M = 0.172, SE = 0.020) and CS- (M = 0.124, SE = 0.017) in God believers (*p* = 0.041, Figure 2). On the other hand, no significant difference was reported for no God believers between CS+ and CS- (*p* = 1.000). No further significant results were identified for extinction (refer to Supplementary Table 4 for details).

Finally, no correlations were found for both groups between State and Trait anxiety with the mean differential SCRs associated to the three sessions (i.e., habituation, conditioning, and extinction). See Table 2 for the details.

**Table 2:** Spearman correlation rank outputs between STAI scores and SCR scores across the three sessions of the Pavlovian conditioning task for the two groups of participants.

	**Session**	**STAI-Y1**	**STAI-Y2**
Non-believers in God	Habituation	R = 0.150	R = −0.022
		*P* = 0.453	*P* = 0.913
	Conditioning	R = 0.231	R = −0.057
		*P* = 0.246	*P* = 0.777
	Extinction	R = 0.160	R = −0.087
		*P* = 0.424	*P* = 0.665
God believers	Habituation	R = −0.016	R = 0.168
		*P* = 0.936	*P* = 0.400
	Conditioning	R = 0.096	R = −0.041
		*P* = 0.630	*P* = 0.839
	Extinction	R = −0.012	R = −0.122
		*P* = 0.950	*P* = 0.542

STAI-Y1, State Trait Anxiety Inventory, Form Y, Subscale 1; STAI-Y1, State Trait Anxiety Inventory, Form Y2, Subscale 2.

### Fear stimulus ratings

A significant main effect of the factor Session was found [*F*_(2, 104)_ = 13.29, *p* < 0.001 η*p*^2^ = 0.203]. *Post-hoc* comparisons show a significant difference (*p* < 0.001) between *Habituation* (*M* = 4.185, *SE* = 0.314) and *Conditioning* (*M* = 5.407, *SE* = 0.226), as well as (*p* < 0.001) between *Conditioning* and *Extinction* (*M* = 4.481, *SE* = 0.337). Furthermore, the main effect of the factor Stimulus was significant [*F*_(1, 52)_ = 21.91, *p* < 0.001 η*p*^2^ = 0.296], with an higher score (*M* = 5.209, *SE* = 0.279) in response to the CS+ compared to the CS- (*M* = 4.172, *SE* = 0.307). Moreover, a significant Stimulus x Session interaction was revealed [*F*_(2, 104)_ = 22.70, *p* < 0.001 η*p*^2^ = 0.303]. *Post-hoc* comparisons document a significant difference between CS+ (*M* = 6.555, *SE* = 0.305) and CS- (*M* = 4.259, *SE* = 0.357) in the Conditioning session (*p* < 0.001). Additionally, a significant difference was found between CS+ (*M* = 4.963, *SE* = 0.363) and CS- (*M* = 4.00, *SE* = 0.348) and CS- for the extinction session (*p* = 0.004). No significant difference was found between CS+ and CS- was found for the habituation session (*p* = 1.000). No further significant results were found (see Supplementary Table 5 for details). Finally, no correlations were found for both groups between State and Trait anxiety with the mean differential FSR associated to the three sessions (i.e., habituation, conditioning, and extinction). See Table 3 for the details.

**Table 3:** Spearman correlation rank outputs between STAI scores and FSR scores across the three sessions of the Pavlovian conditioning task for the two groups of participants.

	**Session**	**STAI-Y1**	**STAI-Y2**
God non-believers	Habituation	R = −0.103	R = −0.121
		*P* = 0.606	*P* = 0.546
	Conditioning	R = −0.037	R = 0.061
		*P* = 0.853	*P* = 0.759
	Extinction	R = −0.347	R = −0.343
		*P* = 0.075	*P* = 0.079
God believers	Habituation	R = −0.045	R = 0.249
		*P* = 0.823	*P* = 0.210
	Conditioning	R = 0.364	R = 0.019
		*P* = 0.061	*P* = 0.923
	Extinction	R = 0.046	R = 0.054
		*P* = 0.818	*P* = 0.786

STAI-Y1, State Trait Anxiety Inventory, Form Y, Subscale 1; STAI-Y1, State Trait Anxiety Inventory, Form Y2, Subscale 2.

## Discussion

In this study, we aimed to investigate whether belief in the existence of God can affect the ability to process fear. We employed a VR-version of Pavlovian fear conditioning paradigm, a widely used translational protocol for the experimental study of mechanisms that underlie pathological fear and anxiety (9, 28–30). A strength of our protocol is that, unlike physical stimuli such as electrical shocks, VR-based stimuli offer the advantage of not requiring individual calibration to accommodate participants' physical differences. This allows researchers to investigate fear responses without inducing discomfort, thereby improving the reliability and interpretability of the findings. In contrast, paradigms employing electrical shocks as the US often face challenges due to variability in physical discomfort sensitivity among participants. Our protocol avoids these limitations by eliminating physical discomfort-inducing stimuli altogether [see also (22) for a discussion]. Moreover, the lower volume of the screaming voice [i.e., 80 dB instead of the traditional 95 dB; see (31)] reduces the aversive impact of the loud noise itself. This demonstrates that the VR paradigm can effectively induce fear conditioning even without traditional unconditioned stimuli (US), such as electrical shocks or loud audio stimuli, as used in Shechner et al. (31). Finally, the SCR pattern in the current study can be interpreted as a “fear response *per se*,” as the persistence of higher SCR to the CS+ (even when the US is omitted) is indicative of fear memory and challenges in inhibitory learning, not just heightened arousal.

Our findings suggest a reduced capacity to extinguish fear in individuals who believe in God compared to non-believers. This conclusion is supported by the higher SCR to the CS+ compared to CS- in God believers during the extinction session. In contrast, no group differences were observed in SCR during the fear conditioning session. This indicates that both groups effectively acquired the association between the CS+ and the US. Moreover, our study found no significant differences in state or trait anxiety between the two groups, nor any correlation between these anxiety measures and the implicit (SCR) and explicit (FSR) measures of fear conditioning. These results suggest that the observed group differences are independent of participants' trait or state anxiety levels. Evidence suggests that anxiety is psychologically and neuronally distinct from fear processing (32, 33), although some overlap between the two has been documented (32–34). This suggests that belief in the existence of God may specifically involve the neural circuitry associated with fear (11), with only minimal involvement of the neural network responsible for anxiety (33). Additionally, no group difference was found in explicit measures (i.e., FSR), with both groups showing higher ratings for the CS+ compared to CS- in both the Conditioning and extinction sessions. This indicates that the influence of belief in God on fear conditioning appears to be limited to the neurovegetative component, rather than affecting conscious, explicit fear responses.

In a Pavlovian fear conditioning task, a neutral stimulus (the CS) is repeatedly paired with an aversive stimulus, such as a shock (the US). Over time, the individual learns to associate the CS with the US, leading to a fear response (such as an increased SCR), whenever the CS is presented. During the extinction phase, the individual is exposed to the CS without the US, indicating that the CS no longer predicts the US. If the fear response persists despite repeated presentations of the CS without the US, it suggests that the fear remains, indicating that the association has not been fully extinguished. The preservation of the neurovegetative fear response in the extinction session could be interpreted as a challenge for God believers in relearning inhibitory process that is expected to occur during fear extinction (35).

Difficulties in inhibiting fear memory traces and in extinguishing fear have been linked to poor stress management (36). As anticipated, some evidence identifies religiosity and spirituality as valuable coping resource (37). However, the poor stress management hypothesis also aligns with studies suggesting that religiosity and spirituality can amplify perceived threat and stress (4, 52).

Although we did not directly measure the degree of participants' religiosity, which is a limitation, our results are line with the interpretation proposed by Winterowd et al. (4) and by Carlozzi et al. (52). Specifically, the failure in fear extinction observed in God believing group supports the hypothesis of a heightened perceived threat and stress in these individuals [for a comprehensive review on the detrimental effects of stress on fear extinction learning see also Maren (38)]. Moreover, our results are aligned with the recent result by Ferguson et al. (11) linking religiosity and spirituality with the neural circuit of fear conditioning and extinction learning (13), and with evidence that religiosity increases under threat (39). Finally, the current results may reflect differences in the cognitive profiles of the two groups of participants. For example, Lindeman and Lipsanen (53) found lower analytical thinking in religious, compared to non-religious individuals. However, to the best of our knowledge, there is no evidence associating this cognitive skill with fear extinction learning. Nonetheless, it might be plausible to expect that a person who can think analytically might be better able to understand and predict when a fear-inducing stimulus is no longer present, potentially aiding in the fear extinction process.

Given that the difficulty in extinguishing fear can be a risk and/or predisposing factor in the genesis of several psychopathological conditions such as post-traumatic stress disorder (40) and anxiety (51), the current results suggest a potentially detrimental role of religiosity in the context of these conditions. This implies that religiosity could be a relevant variable affecting the process of fear recovery following traumatic experiences. While existing literature highlights the potential benefits of religiosity in alleviating mental health disorders (41), particularly by providing emotional support and coping mechanisms, there is also evidence suggesting that certain religious ideologies may be linked to mental distress (42). Our findings align more closely with the latter perspective, indicating that religiosity could pose challenges for exposure-based therapies. Given these mixed outcomes, a systematic investigation that not only explores the potential benefits and drawbacks of religiosity but also considers the differences across various religious traditions is crucial for advancing our understanding in this area. Such research would help identify specific factors that might influence how religiosity interacts with therapeutic approaches.

Based on these findings, future investigations could build upon this study by exploring the connection between fear conditioning and religiosity using questionnaires-based methods. For instance, it would be relevant incorporating widely accepted belief-in-God scales, which reflect a spectrum from atheism to theism [e.g., (43, 58)]. Including these scales would provide a test for the curvilinear relationship between religiosity and fear processing, a well-established finding in relation to mental wellbeing (44) and prejudice (45). Furthermore, it would be intriguing to investigate potential differences between various types of religions, as fear can be perceived both as a source of comfort in fearful situations and as a potential enhancer of fear, and a limitation of our study is our focus on Catholic believers. In this regard, it would be interesting to explore the extent to which the current results apply to individuals of other religious affiliations and to the various facets of religiosity, including fear of crime (46), fear of death, and fear of the unknown. Moreover, it would be intriguing to explicitly investigate the tendency to forgiving, which is encouraged in Catholic spirituality, as the current study reports a difficulty of God believers in extinguishing fear, which might reflect interaction between religious beliefs and existential fears.

Since our participants were tested using a Pavlovian fear conditioning protocol that did not include stimuli and conditions explicitly relevant to religiosity, future investigations could incorporate these aspects into the protocols. This would help assess the generalizability of these results within the context of religiosity. Moreover, it would be of interest extending the current investigation by including the extinction recall and fear renewal phases of this task and including neuroimaging methods [e.g., see (47)] to outline respective neural correlates. Adding extinction recall would allow for assessing whether such difference in fear extinction between God believers and non-believers is maintained over time. This could reinforce the conclusion that belief in God is associated with sustained fear responses, highlighting its implications for exposure-based therapies. Moreover, adding fear renewal phases would provide valuable insights into the context dependency and persistence of fear extinction differences between the two examined groups of participants. This would provide a more comprehensive picture of the stability and context sensitivity of these differences over time.

Finally, exploring variables related to personality traits, emotion regulation, alexithymia, coping styles, or cultural factors could provide additional insights, given their relevance to risk taking behavior (48, 55), fear conditioning (17), mood (60), and ideological thought (49). Examining these factors could deepen our understanding of the psychophysiological mechanisms underlying the complex relationship between religiosity and fear processing.

## Data Availability

The data analyzed in this study is subject to the following licenses/restrictions: Data will be provided following motivated request. Requests to access these datasets should be directed to cvicario@unime.it.
