# Supplementary material for: The power of belief? Evidence of reduced fear extinction learning in Catholic God believers
Source: Front Public Health. 2025 Jan 7;12:1509388. doi: 10.3389/fpubh.2024.1509388 (PMC11748304; doi:10.3389/fpubh.2024.1509388)
Supplement: Supplementary file 1 [file Supplementary_file_1.docx]

**Supplemental Materials**

Skin Conductance response

Habituation

| Repeated Measures Analysis of Variance with Effect Sizes and Powers (Spreadsheet1) Sigma-restricted parameterization Effective hypothesis decomposition | | | | | | | | |
| --- | --- | --- | --- | --- | --- | --- | --- | --- |
|  | **SS** | **Degr. of - Freedom** | **MS** | **F** | **p** | **Partial eta-squared** | **Non-centrality** | **Observed power (alpha=0,05)** |
| **Intercept** | 17,00045 | 1 | 17,00045 | 117,6064 | 0,000000 | 0,693408 | 117,6064 | 1,000000 |
| **Believer** | 0,31972 | 1 | 0,31972 | 2,2118 | 0,142998 | 0,040799 | 2,2118 | 0,308788 |
| **Error** | 7,51679 | 52 | 0,14455 |  |  |  |  |  |
| **CS** | 0,08753 | 1 | 0,08753 | 1,7759 | 0,188467 | 0,033024 | 1,7759 | 0,257775 |
| **CS*Believer** | 0,08466 | 1 | 0,08466 | 1,7178 | 0,195732 | 0,031979 | 1,7178 | 0,250915 |
| **Error** | 2,56288 | 52 | 0,04929 |  |  |  |  |  |
| **TRIALS** | 1,95750 | 3 | 0,65250 | 17,5196 | 0,000000 | 0,252010 | 52,5589 | 0,999998 |
| **TRIALS*Believer** | 0,10769 | 3 | 0,03590 | 0,9638 | 0,411434 | 0,018198 | 2,8915 | 0,259603 |
| **Error** | 5,81005 | 156 | 0,03724 |  |  |  |  |  |
| **CS*TRIALS** | 0,22093 | 3 | 0,07364 | 2,0303 | 0,111897 | 0,037578 | 6,0910 | 0,513623 |
| **CS*TRIALS*Believer** | 0,11448 | 3 | 0,03816 | 1,0521 | 0,371305 | 0,019832 | 3,1563 | 0,281223 |
| **Error** | 5,65825 | 156 | 0,03627 |  |  |  |  |  |

**Table S1.**

Acquisition

| Repeated Measures Analysis of Variance with Effect Sizes and Powers (Spreadsheet1) Sigma-restricted parameterization Effective hypothesis decomposition | | | | | | | | |
| --- | --- | --- | --- | --- | --- | --- | --- | --- |
|  | **SS** | **Degr. of - Freedom** | **MS** | **F** | **p** | **Partial eta-squared** | **Non-centrality** | **Observed power (alpha=0,05)** |
| **Intercept** | 96,61434 | 1 | 96,61434 | 168,0451 | 0,000000 | 0,763685 | 168,0451 | 1,000000 |
| **Believer** | 1,49332 | 1 | 1,49332 | 2,5974 | 0,113094 | 0,047574 | 2,5974 | 0,352890 |
| **Error** | 29,89641 | 52 | 0,57493 |  |  |  |  |  |
| **CS** | 8,47156 | 1 | 8,47156 | 52,3514 | 0,000000 | 0,501684 | 52,3514 | 1,000000 |
| **CS*Believer** | 0,25797 | 1 | 0,25797 | 1,5942 | 0,212365 | 0,029745 | 1,5942 | 0,236263 |
| **Error** | 8,41470 | 52 | 0,16182 |  |  |  |  |  |
| **EL** | 0,75901 | 1 | 0,75901 | 13,9173 | 0,000474 | 0,211132 | 13,9173 | 0,955517 |
| **EL*Believer** | 0,03359 | 1 | 0,03359 | 0,6159 | 0,436126 | 0,011706 | 0,6159 | 0,120271 |
| **Error** | 2,83595 | 52 | 0,05454 |  |  |  |  |  |
| **TRIALS** | 0,44693 | 4 | 0,11173 | 3,1053 | 0,016465 | 0,056353 | 12,4214 | 0,807651 |
| **TRIALS*Believer** | 0,13331 | 4 | 0,03333 | 0,9262 | 0,449590 | 0,017501 | 3,7050 | 0,291193 |
| **Error** | 7,48401 | 208 | 0,03598 |  |  |  |  |  |
| **CS*EL** | 0,16064 | 1 | 0,16064 | 2,6488 | 0,109676 | 0,048469 | 2,6488 | 0,358675 |
| **CS*EL*Believer** | 0,00561 | 1 | 0,00561 | 0,0926 | 0,762136 | 0,001777 | 0,0926 | 0,060282 |
| **Error** | 3,15362 | 52 | 0,06065 |  |  |  |  |  |
| **CS*TRIALS** | 0,15116 | 4 | 0,03779 | 1,0612 | 0,376749 | 0,020000 | 4,2450 | 0,331519 |
| **CS*TRIALS*Believer** | 0,15530 | 4 | 0,03883 | 1,0903 | 0,362321 | 0,020538 | 4,3614 | 0,340217 |
| **Error** | 7,40666 | 208 | 0,03561 |  |  |  |  |  |
| **EL*TRIALS** | 0,06046 | 4 | 0,01512 | 0,4008 | 0,807971 | 0,007648 | 1,6031 | 0,141862 |
| **EL*TRIALS*Believer** | 0,05134 | 4 | 0,01284 | 0,3403 | 0,850551 | 0,006502 | 1,3612 | 0,126332 |
| **Error** | 7,84532 | 208 | 0,03772 |  |  |  |  |  |
| **CS*EL*TRIALS** | 0,05287 | 4 | 0,01322 | 0,3329 | 0,855623 | 0,006361 | 1,3316 | 0,124467 |
| **CS*EL*TRIALS*Believer** | 0,08770 | 4 | 0,02192 | 0,5522 | 0,697655 | 0,010507 | 2,2086 | 0,182680 |
| **Error** | 8,25893 | 208 | 0,03971 |  |  |  |  |  |

**Table S2.**

Extinction

| Repeated Measures Analysis of Variance with Effect Sizes and Powers (Spreadsheet1) Sigma-restricted parameterization Effective hypothesis decomposition | | | | | | | | |
| --- | --- | --- | --- | --- | --- | --- | --- | --- |
|  | **SS** | **Degr. of - Freedom** | **MS** | **F** | **p** | **Partial eta-squared** | **Non-centrality** | **Observed power (alpha=0,05)** |
| **Intercept** | 17,95387 | 1 | 17,95387 | 116,3794 | 0,000000 | 0,691174 | 116,3794 | 1,000000 |
| **Believer** | 0,40510 | 1 | 0,40510 | 2,6259 | 0,111183 | 0,048070 | 2,6259 | 0,356102 |
| **Error** | 8,02205 | 52 | 0,15427 |  |  |  |  |  |
| **CS** | 0,13159 | 1 | 0,13159 | 3,3522 | 0,072847 | 0,060561 | 3,3522 | 0,435387 |
| **CS*Believer** | 0,18009 | 1 | 0,18009 | 4,5879 | 0,036903 | 0,081075 | 4,5879 | 0,556608 |
| **Error** | 2,04119 | 52 | 0,03925 |  |  |  |  |  |
| **EL** | 0,10785 | 1 | 0,10785 | 3,1541 | 0,081587 | 0,057187 | 3,1541 | 0,414292 |
| **EL*Believer** | 0,00605 | 1 | 0,00605 | 0,1770 | 0,675695 | 0,003392 | 0,1770 | 0,069759 |
| **Error** | 1,77807 | 52 | 0,03419 |  |  |  |  |  |
| **TRIALS** | 0,04560 | 4 | 0,01140 | 0,4235 | 0,791609 | 0,008078 | 1,6939 | 0,147817 |
| **TRIALS*Believer** | 0,18528 | 4 | 0,04632 | 1,7207 | 0,146624 | 0,032031 | 6,8829 | 0,521490 |
| **Error** | 5,59903 | 208 | 0,02692 |  |  |  |  |  |
| **CS*EL** | 0,00092 | 1 | 0,00092 | 0,0361 | 0,850149 | 0,000693 | 0,0361 | 0,053989 |
| **CS*EL*Believer** | 0,00135 | 1 | 0,00135 | 0,0528 | 0,819085 | 0,001015 | 0,0528 | 0,055854 |
| **Error** | 1,32482 | 52 | 0,02548 |  |  |  |  |  |
| **CS*TRIALS** | 0,03247 | 4 | 0,00812 | 0,2743 | 0,894351 | 0,005247 | 1,0971 | 0,109965 |
| **CS*TRIALS*Believer** | 0,07616 | 4 | 0,01904 | 0,6433 | 0,632201 | 0,012220 | 2,5732 | 0,208337 |
| **Error** | 6,15595 | 208 | 0,02960 |  |  |  |  |  |
| **EL*TRIALS** | 0,11380 | 4 | 0,02845 | 0,9878 | 0,415163 | 0,018642 | 3,9512 | 0,309565 |
| **EL*TRIALS*Believer** | 0,02666 | 4 | 0,00667 | 0,2314 | 0,920506 | 0,004431 | 0,9257 | 0,099697 |
| **Error** | 5,99090 | 208 | 0,02880 |  |  |  |  |  |
| **CS*EL*TRIALS** | 0,21085 | 4 | 0,05271 | 1,4374 | 0,222797 | 0,026899 | 5,7497 | 0,442441 |
| **CS*EL*TRIALS*Believer** | 0,10411 | 4 | 0,02603 | 0,7098 | 0,586097 | 0,013465 | 2,8390 | 0,227434 |
| **Error** | 7,62756 | 208 | 0,03667 |  |  |  |  |  |

**Table S3**

| **Bonferroni test; variable DV_1 (Spreadsheet7) Probabilities for Post Hoc Tests Error: Between; Within; Pooled MSE = ,00968, df = 76,853** | | | | | | |
| --- | --- | --- | --- | --- | --- | --- |
|  | **Believer** | **CS** | **{1} - ,10769** | **{2} - ,11144** | **{3} - ,17225** | **{4} - ,12435** |
| **1** | **No** | **CS+** |  | **1,000000** | **0,109649** | **1,000000** |
| **2** | **No** | **CS-** | **1,000000** |  | **0,155527** | **1,000000** |
| **3** | **Yes** | **CS+** | **0,109649** | **0,155527** |  | **0,041886** |
| **4** | **Yes** | **CS-** | **1,000000** | **1,000000** | **0,041886** |  |

**Table S4**

Fear Stimulus rating

| Repeated Measures Analysis of Variance with Effect Sizes and Powers (Spreadsheet3) Sigma-restricted parameterization Effective hypothesis decomposition | | | | | | | | |
| --- | --- | --- | --- | --- | --- | --- | --- | --- |
|  | **SS** | **Degr. of - Freedom** | **MS** | **F** | **p** | **Partial eta-squared** | **Non-centrality** | **Observed power (alpha=0,05)** |
| **Intercept** | 7130,864 | 1 | 7130,864 | 296,9578 | 0,000000 | 0,850985 | 296,9578 | 1,000000 |
| **Believer** | 4,457 | 1 | 4,457 | 0,1856 | 0,668386 | 0,003557 | 0,1856 | 0,070729 |
| **Error** | 1248,679 | 52 | 24,013 |  |  |  |  |  |
| **HAE** | 87,802 | 2 | 43,901 | 13,2987 | 0,000007 | 0,203660 | 26,5974 | 0,997245 |
| **HAE*Believer** | 3,877 | 2 | 1,938 | 0,5871 | 0,557742 | 0,011165 | 1,1743 | 0,145370 |
| **Error** | 343,321 | 104 | 3,301 |  |  |  |  |  |
| **CS** | 87,111 | 1 | 87,111 | 21,9196 | 0,000021 | 0,296533 | 21,9196 | 0,995779 |
| **CS*Believer** | 3,568 | 1 | 3,568 | 0,8978 | 0,347759 | 0,016972 | 0,8978 | 0,153462 |
| **Error** | 206,654 | 52 | 3,974 |  |  |  |  |  |
| **HAE*CS** | 80,889 | 2 | 40,444 | 22,7030 | 0,000000 | 0,303910 | 45,4060 | 0,999992 |
| **HAE*CS*Believer** | 1,506 | 2 | 0,753 | 0,4227 | 0,656372 | 0,008064 | 0,8455 | 0,116979 |
| **Error** | 185,272 | 104 | 1,781 |  |  |  |  |  |

**Table S4**
